# Midline Venous Catheter vs Peripherally Inserted Central Catheter for Intravenous Therapy: A Randomized Clinical Trial

**DOI:** 10.1001/jamanetworkopen.2025.1258

**Published:** 2025-03-20

**Authors:** Ahmed Bentridi, Marie-France Giroux, Gilles Soulez, Louis Bouchard, Pierre Perreault, Audrey Chouinard, Marc Dorais, Ricardo Do Amaral, Pascaline Bernier, Eric Therasse

**Affiliations:** 1 Department of Vascular and Interventional Radiology, Centre Hospitalier de l’Université de Montréal, Montréal, Québec, Canada; 2 Now with Department of Radiology, Hôpital du Sacré-Coeur de Montréal, Montréal, Québec, Canada; 3 Department of Nursing, Centre Hospitalier de l’Université de Montréal, Montréal, Québec, Canada; 4 StatSciences Inc, Notre-Dame-de-l’Île-Perrot, Quebec, Canada; 5 Department of Pharmacy, Centre Hospitalier de l’Université de Montréal, Montréal, Québec, Canada

## Abstract

**Importance:**

Peripherally inserted central catheters (PICCs) are frequently used for peripheral intravenous therapy (IVT) that could be administered through a peripheral midline venous catheter (MVC).

**Objective:**

To assess the noninferiority of MVCs compared with PICCs as a reliable vascular access for peripheral IVT and blood draws for IVT that does not require a central VC.

**Design, Setting, and Participants:**

This randomized clinical trial was conducted in a single tertiary care center from September 2018 to March 2022. Participants were all consecutive adult patients who were referred for PICC and eligible for MVC. Patients likely to require a central VC (those in the critical care unit, those with kidney failure, or those requiring a multilumen VC) were excluded. Analyses were based on the evaluable population.

**Interventions:**

Participants were randomized 1:1 to either MVC or PICC. For the MVC group, a 20-cm-long, 4F (French), single-lumen MVC without a valve was used without fluoroscopic assistance. For the PICC group, a 4F, single-lumen PICC without a valve was positioned under fluoroscopy at the cavoatrial junction.

**Main Outcomes and Measures:**

The primary outcome was the percentage of patients without VC-related adverse events or dysfunctions requiring medical intervention during follow-up. A noninferiority test was performed to compare the proportion of adverse events or dysfunctions between the MVC and PICC groups. A noninferiority margin was set at 10% and a 5% 1-sided significance level.

**Results:**

Of the 6821 patients referred to the tertiary care center for PICC insertion, 294 (180 males [61.2%]; median [IQR] age, 56.3 [38.2-66.7] years) were randomized to receive MVCs (n = 146) or PICCs (n = 148); 135 and 137 participants, respectively, were included in data analysis after exclusion of those who did not complete follow-up. Ninety of 135 patients (66.7%) in the MVC group and 128 of 137 (93.4%) in the PICC group were without VC-related adverse event or dysfunction. The noninferiority of MVC could not be demonstrated (*P* > .99 for noninferiority).

**Conclusions and Relevance:**

In this randomized clinical trial, MVCs were associated with a significantly higher percentage of patients with VC-related adverse events or dysfunctions and could not be demonstrated as a noninferior alternative to PICCs.

**Trial Registration:**

ClinicalTrials.gov Identifier: NCT03502980

## Key Points

**Question:**

Is a midline venous catheter (MVC) a noninferior alternative to a peripherally inserted central catheter (PICC) for peripheral intravenous therapy in a tertiary care center?

**Findings:**

In this randomized clinical trial involving 294 patients, the percentage of patients without venous catheter–related adverse event or dysfunction was significantly lower in the MVC group than in the PICC group, and the noninferiority of MVC could not be demonstrated.

**Meaning:**

The findings of this trial indicate that MVC was not a noninferior alternative to PICC for peripheral intravenous therapy in a tertiary care center.

## Teaser

This randomized clinical trial compares the adverse events or dysfunctions occurring with use of midline venous catheters vs peripherally inserted central catheters (PICCs) among a select group of adult patients in a tertiary care center.

## Introduction

Peripheral venous catheters (VCs) are appropriate for intravenous therapy (IVT) with nonirritating medication that does not require a central access.^1,2^ Central VCs are generally used for IVT that requires blood dilution in a large vein, such as the superior vena cava. Peripherally inserted central catheters (PICCs) are central VCs inserted through a peripheral vein, avoiding complications associated with a central vein puncture. In many tertiary care centers, PICCs have become popular and are now often used even for IVT that does not need a central VC.^2^ However, PICC insertion may require some guidance, which adds to the cost and time needed in comparison with the midline venous catheter (MVC).^3,4,5,6^

According to the Infusion Nurses Society 2016 Therapy Standards of Practice,^1^ an MVC should be considered when the anticipated duration of treatment ranges from 1 to 4 weeks. The 2016 Michigan Appropriateness Guide for Intravenous Catheters states that an MVC is preferred over a PICC for peripherally compatible infusate of 6 to 14 days.^5^ However, these recommendations are mostly based on expert opinions in systematic reviews of cohort studies.^5,7^ Despite lower cost and better accessibility of MVCs compared with PICCs in ambulatory patients, there is little evidence in the literature suggesting that MVCs are a noninferior alternative to PICCs for peripheral IVT in a tertiary care center.^8,9,10,11^

We hypothesized that, in a tertiary care setting, MVCs could have an advantage to replace PICCs for short-term peripheral IVT. Hence, the primary objective of this study was to assess the noninferiority of MVCs compared with PICCs as a reliable vascular access for peripheral IVT and blood draws for 1 to 4 weeks of IVT that does not require a central VC.

## Methods

### Study Design and Population

This prospective, open-label, parallel-group, noninferiority randomized clinical trial was performed in a single 700-bed, tertiary care university hospital from September 2018 to March 2022. The full trial protocol is provided in Supplement 1. This protocol was approved by the Centre de Recherche du Centre Hospitalier de l'Université de Montréal Ethics Committee. Written informed consent was obtained from all patients. We followed the Consolidated Standards of Reporting Trials (CONSORT) reporting guideline.

Patient demographic data were obtained from medical records. Race and ethnicity (American Indian or Alaska Native, Asian, Black or African American, Hispanic, Native Hawaiian or Other Pacific Islander, North African, White, and other [Bengali, Indo-European, Persian]) were observed by the investigator and collected in this study for descriptive purposes.

All consecutive adult patients referred to the tertiary care center’s radiology department for PICC insertion who were eligible for MVC according to recommendations of the Infusion Nurses Society 2016 Therapy Standards of Practice were considered for inclusion.^1^ Exclusion criteria were (1) ineligibility for MVC due to expected IVT for fewer than 6 days or more than 30 days, vesicant IVT (pH <5 and pH >9, osmolarity >900 mOsm/L, parenteral nutrition, chemotherapy, potassium >40 mEq/L [to convert to mmol/L, multiply by 1], and vasopressors), kidney disease requiring vein preservation (estimated glomerular filtration rate [eGFR] <30 mL/min/1.73 m^2^), preexisting venous thrombosis or known hypercoagulable state, inability to provide informed consent, and (2) likely need for a central VC at follow-up due to requirement for VC with multiple lumens (as indicated on the physician’s PICC request) and being in a critical care unit. In September 2019, the trial protocol was amended to exclude patients with difficult blood draws who required repeated blood sampling (as indicated on the request) because of concerns about partial occlusion in the MVC group. During the COVID-19 pandemic, patients with positive COVID-19 test results or evocative clinical symptoms were also excluded. Given the slow enrollment rate during the COVID-19 pandemic, the trial protocol was also amended to allow inclusion of patients with eGFR of less than 30 mL/min/1.73 m^2^.

### Randomization and Masking

Randomization was stratified according to the indication for VC: for antibiotics vs other IVT as well as the presence vs absence of cystic fibrosis, as these 2 factors may be associated with thrombosis exposure.^6,12,13^ Randomization allocation tables were generated using package blockrand in R, version 4.1.0 (R Project for Statistical Computing) and uploaded into a REDCap database (Vanderbilt University). Allocation was done by blocked randomization, with block sizes randomly varying between 2 and 4. Patients, physician, and research staff were all aware of the treatment group assignment (MVC or PICC).

### Devices and Insertion Procedure

MVCs and PICCs were inserted by either interventional radiologists, fellows, residents, or accredited radiological technologists in the same angiography suites. Sterile techniques were used with operators wearing a mask, a cap, and sterile gown and gloves. The puncture site preparation, with a 2% chlorhexidine and 70% alcohol solution, and draping were similar for both groups. The basilic or brachial vein was punctured above the elbow joint using ultrasonographic guidance. For the MVC group, a 20-cm-long, 4F (French), single-lumen MVC without valve (PowerMidline Catheter; BD) was inserted without fluoroscopic assistance. The distance between the axilla and the puncture site was measured and, when needed, the catheter’s tip was cut so that it did not end into the curved portion of the axillary vein. For the PICC group, a 4F, single-lumen PICC without a valve (PowerPICC Catheter; BD) was positioned under fluoroscopy at the cavoatrial junction. Catheter fixation and dressing were similar for both groups as per standard protocol. No anticoagulant was administered even if the VC was not used for continuous infusion.

### Patient Follow-Up

Patients were followed up until 7 weeks after VC insertion or until 1 week after removal. Safety and efficacy assessments were achieved through weekly communications between the research assistant and the patient’s health care practitioner or between the research assistant and the patient. Prevention and treatment of VC-related infections were provided according to standard guidelines.^14,15^ Erythema, induration, and purulent discharge at the site of catheter insertion were reported as catheter-related soft-tissue infections. Patients who reported local pain, edema, or superficial vein enlargement were referred for ultrasonography. There was no routine imaging follow-up.

### Study Outcomes

The primary outcome was the percentage of patients without VC-related adverse events or dysfunctions requiring medical intervention during follow-up. Adverse events were defined as suspected or confirmed catheter-related bloodstream or local infections; deep or superficial vein thrombosis infiltration; and VC-related pain, bleeding, or death. VC dysfunctions were defined as accidental withdrawal or migration, leakage or fracture, and complete occlusion (defined as the impossibility to inject treatments) or partial occlusion (defined as the impossibility to draw blood) in patients with difficult blood draws.^16^ Medical interventions included but were not limited to VC removal, repositioning, or replacement and drug administration. Secondary outcomes were the percentage of patients with each of the aforementioned VC-related adverse events or dysfunctions, the number of these VC-related adverse events and dysfunctions per 1000 catheter-days, survival without these VC-related adverse events and dysfunctions, the VC procedure duration, the need for additional measures to insert the VC such as contrast injection and fluoroscopy, the percentage of patients who required another VC, and the percentage of patients with premature VC removal due to dysfunction or adverse event.

### Statistical Analysis

Sample size was based on the primary outcome. Based on a previous audit conducted at the medical center and based on the existing literature,^6,10^ we considered that 75% of patients with a PICC would be free from VC-related adverse events or dysfunctions. With a noninferiority margin set at 10% (considered acceptable given that MVCs were more accessible and less expensive than PICCs at the radiology department), we estimated that 464 patients would be needed to provide 80% power to exclude a difference of more than 10% in favor of the PICC group over the MVC group, with a 5% 1-sided significance level. To compensate for patients lost to follow-up, we increased the total sample size by 10% to 510 patients. Differences between groups were assessed using Pearson χ^2^ test or Fisher exact test for categorical variables and Wilcoxon-Mann-Whitney test for continuous variables. For the primary end point, a noninferiority test comparing the proportion of adverse events or dysfunctions was calculated to determine whether the MVC group was noninferior to the PICC group. For all other end points, differences between groups were tested for superiority. Poisson regression was performed to compare rates of events per 1000 catheter-days. The percentage of patients without adverse events or dysfunctions was calculated using Kaplan-Meier estimators, and a log-rank test was used to compare both groups.

All statistical analyses of the evaluable population were performed using SAS, version 9.4 (SAS Institute Inc). A *P* < .05 was considered statistically significant.

## Results

**Figure 1.**
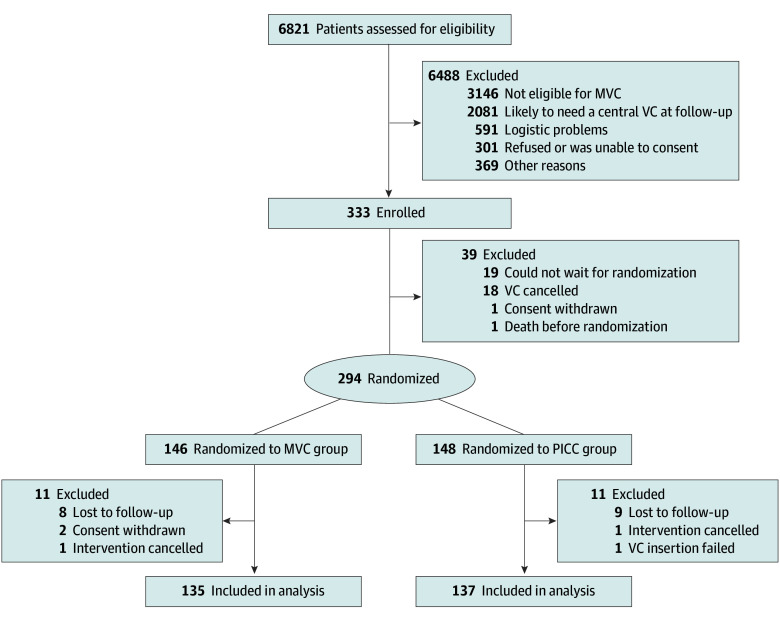
Flowchart of Study Population Follow-up was available for 272 (92.5%) of 294 randomized patients. MVC indicates midline venous catheter; PICC, peripherally inserted central catheter; and VC, venous catheter.

Between September 2018 and March 2022, a total of 6821 patients referred for PICC were screened, of whom 333 met the inclusion criteria and 294 (114 females [38.8%] and 180 males [61.2%]; median [IQR] age, 56.3 [38.2-66.7] years) were randomized to the MVC group (n = 146) or the PICC group (n = 148) (Figure 1; Table 1). The main reason for study exclusion was patients’ ineligibility for MVC for IVT (n = 3146 of 6821 patients [46.1%]), including those with a need for a central VC (n = 2155 [31.6%]), expected IVT for fewer than 6 days or more than 30 days (n = 468 [6.9%]), eGFR less than 30 mL/min/1.73 m^2^ (n = 392 [5.7%]), and other medical criteria (n = 131 [1.9%]). In addition, 2081 patients (30.5%) who were likely to need a central VC at follow-up were excluded, of whom 1118 (16.4%) required a multilumen VC, 576 (8.4%) were in the intensive care unit, and 387 (5.7%) had difficult blood draws. Due to logistical problems, mainly during the COVID-19 pandemic, 591 patients (8.7%) were excluded. Study enrollment was interrupted prematurely because of low enrollment due to the high exclusion rate and the impossibility of recruiting patients during the COVID-19 pandemic.

**Table 1.** Patient Demographics and Venous Access Characteristics

Characteristic	Patients, No. (%)	
	MVC (n = 146)	PICC (n = 148)
Age, median (IQR), y	56.3 (38.9-66.8)	56.7 (38.0-66.4)
Sex		
Male	96 (65.7)	84 (56.8)
Female	50 (34.3)	64 (43.2)
Race and ethnicity^a^		
American Indian or Alaska Native	0	1 (0.7)
Asian	0	1 (0.7)
Black or African American	7 (4.8)	1 (0.7)
Hispanic	1 (0.7)	3 (2.0)
Native Hawaiian or Other Pacific Islander	1 (0.7)	0
North African	4 (2.7)	1 (0.07)
White	130 (89.0)	137 (92.6)
Other^c^	3 (2.1)	4 (2.7)
Cystic fibrosis	38 (26.0)	38 (25.7)
eGFR, median (IQR), mL/min/1.73 m^2^	101.5 (86.0-117.0)	100.5 (81.0-116.0)
Access placement indication		
Antibiotic therapy	141 (96.6)	141 (95.3)
Vancomycin	20 (13.7)	10 (6.8)
Other^b^	5 (3.4)	7 (4.7)
Difficult venous access		
Patients with data	140 (95.9)	146 (98.6)
Patients with difficult venous access	13 (9.3)	20 (13.7)
Patient status		
Inpatient	124 (84.9)	117 (79.0)
Outpatient	22 (15.1)	31 (31.0)
Side of insertion		
Patients with data	136 (93.2)	146 (98.6)
Right side	114 (83.8)	118 (80.8)
Left side	22 (16.2)	28 (19.2)
Operator		
Radiologist	75 (51.3)	71 (48.0)
Fellow or resident	70 (48.0)	76 (51.3)
Accredited radiologic technologist	1 (0.7)	1 (0.7)
Intervention duration		
Patients with data	144 (98.6)	146 (98.6)
Duration, median (IQR), min	35 (26-47)	33 (25-41)
Contrast injection		
Patients with data	140 (95.9)	145 (98.0)
Patients with contrast injection	1 (0.7)	8 (5.5)
Fluoroscopy dose		
Patients with data	140 (95.9)	143 (96.6)
Median (IQR), mGy	0	2.0 (1.0-5.0)
Fluoroscopy time		
Patients with data	140 (95.9)	143 (96.6)
Time, median (IQR), min	0	0.3 (0.1-0.7)

Abbreviations: eGFR, estimated glomerular filtration rate; mGy, milligray; MVC, midline venous catheter; PICC, peripherally inserted central catheter.

^a^ Observed by investigator.

^b^ Other indication included other medications, blood draws, and hydration.

^c^ Other included Bengali, Indo-European, and Persian races and ethnicities.

Baseline characteristics of the MVC and PICC groups are reported in Table 1. Both groups had similar demographic and interventional characteristics. VCs were inserted mostly for antibiotic therapy (282 [95.9%]), and there was a predominance of inpatient status (241 [82.0%]). Cystic fibrosis was prevalent in patients (76 [25.9%]).

Twenty-two of 294 patients (7.5%) did not complete follow-up, leaving 135 patients in the MVC group and 137 in the PICC group for the analyses. Two patients in the PICC group died before the first follow-up and 1 in the MVC group died during the follow-up. There were no VC-related deaths or severe adverse events in either group.

**Figure 2.**
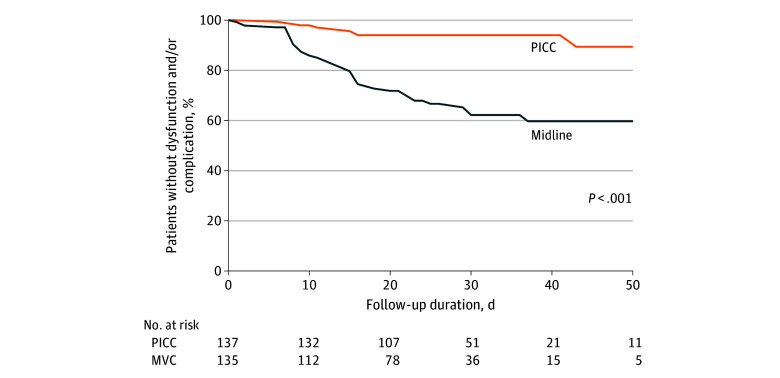
Kaplan-Meyer Analysis of Freedom From Venous Catheter (VC)–Related Adverse Event or Dysfunction At 50 days after VC insertion, 53.7% of patients in the midline venous catheter (MVC) group and 89.4% in the peripherally inserted central catheter (PICC) group were free from VC-related adverse event or dysfunction (*P* < .001).

The outcomes of participants are reported in Table 2. The percentage of patients without VC-related adverse event or dysfunction (primary outcome) was 66.7% (90 of 135) in the MVC group and 93.4% (128 of 137) in the PICC group (*P* > .99 for noninferiority). Patients in the MVC group vs the PICC group had more VC dysfunctions (41 of 135 [30.4%] vs 8 of 137 [5.8%]; *P* < .001) and adverse events (7 of 135 [5.2%] vs 2 of 137 [1.5%]; *P* = .10). VC-related dysfunctions or adverse events per 1000 catheter-days were 17.8 (95% CI, 13.6-22.1) and 2.9 (95% CI, 1.3-4.6) in the MVC and PICC groups, respectively, (*P* < .001). On Kaplan-Meyer analysis, the proportion of patients free from VC-related adverse event or dysfunction at 50 days after VC insertion was 89.3% and 59.7% in the PICC and MVC groups, respectively, (*P* < .001) (Figure 2). In a post hoc analysis excluding partial occlusion (eTable in Supplement 2), the percentage of patients without VC-related adverse event or dysfunction was 88.1% (119 of 135) and 94.2% (129 of 137) in the MVC and PICC groups, respectively, (*P* = .13 for noninferiority).

**Table 2.** Venous Catheter–Related Adverse Events or Dysfunction

	Patients, No. (%)^a^		*P* value^b^
	MVC (n = 135)	PICC (n = 137)	
Patients without VC-related dysfunction or adverse event	90 (66.7)	128 (93.4)	>.99^c^
No. of VC-related dysfunctions or adverse events			
1	27 (20.0)	6 (4.4)	<.001
2	13 (9.6)	3 (2.2)	
3	5 (3.7)	0	
Total No. of VC-related dysfunction or adverse event	68^d^	12^d^	NA
Catheter dwell time			
Median (IQR), d	26 (21-36)	29 (22-37)	.40
Total No. of catheter-days	3813^d^	4070^d^	NA
Per 1000 catheter-days (95% CI)	17.8 (13.6-22.1)	2.9 (1.3-4.6)	<.001
Patients without VC-related dysfunction	94 (69.6)	129 (94.1)	<.001
No. of VC-related dysfunctions	41 (30.4)	8 (5.8)	<.001
1	25 (18.5)	6 (4.4)	<.001
2	12 (8.9)	2 (1.5)	
3	4 (3.0)	0	
Total No. of VC-related dysfunctions	61^d^	10^d^	NA
Per 1000 catheter-days (95% CI)	16.0 (12.0-20.0)	2.5 (0.9-4.0)	<.001
VC migration or accidental withdrawal	4 (3.0)	1 (0.7)	.21
Per 1000 catheter-days (95% CI)	1.0 (0.0-2.1)	0.2 (0.0-0.7)	.19
Complete or partial VC occlusions^e^	37 (27.4)	7 (5.1)	<.001
Per 1000 catheter-days (95% CI)	9.7 (6.6-12.8)	1.7 (0.4-3.0)	<.001
Complete occlusions	7 (5.2)	6 (4.4)	.76
Per 1000 catheter-days (95% CI)	1.8 (0.5-3.2)	1.5 (0.3-2.7)	.69
Partial occlusions	30 (22.2)	1 (0.7)	<.001
Per 1000 catheter-days (95% CI)	7.9 (5.1-10.7)	0.2 (0.0-0.7)	.01
Patients without VC-related adverse event	128 (94.8)	135 (98.5)	NA
Patients with 1 VC-related adverse event	7 (5.2)	2 (1.5)	.10
Total No. of VC-related adverse event	7^d^	2^d^	NA
Per 1000 catheter-days (95% CI)	1.8 (0.5-3.2)	0.5 (0.0-1.2)	.10
With VC infiltrations	1 (0.7)	0	.50
Per 1000 catheter-days (95% CI)	0.3 (0.0-0.8)	0	>.99
With VC bleeding at insertion site	3 (2.2)	0	.12
Per 1000 catheter-days (95% CI)	0.8 (0.0-1.7)	0	>.99
With VC-related infections	1 (0.7)	1 (0.7)	>.99
Per 1000 catheter-days (95% CI)	0.3 (0.0-0.8)	0.2 (0.0-0.7)	.96
With local infections	1 (0.7)	1 (0.7)	>.99
Per 1000 catheter-days (95% CI)	0.3 (0.0-0.8)	0.2 (0.0-0.7)	.96
With bloodstream infections	0	0	NA
Per 1000 catheter-days (95% CI)	0	0	NA
With VC-related thrombophlebitis	2 (1.5)	1 (0.7)	.62
Per 1000 catheter-days (95% CI)	0.5 (0.0-1.3)	0.2 (0.0-0.7)	.54
With deep thrombophlebitis	0	0	NA
Per 1000 catheter-days (95% CI)	0	0	NA
With superficial thrombophlebitis	2 (1.5)	1 (0.7)	.62
Per 1000 catheter-days (95% CI)	0.5 (0.0-1.3)	0.2 (0.0-0.7)	.54

Abbreviations: MVC, midline venous catheter; NA, not applicable; PICC, peripherally inserted central catheter; VC, venous catheter.

^a^ Wilcoxon-Mann-Whitney test was used for comparison of medians. Poisson regression was used for comparison of rates per 1000 catheter-days.

^b^ Calculated with Pearson χ^2^ test, Fisher exact test, or Wilcoxon-Mann-Whitney test, as appropriate.

^c^ Noninferiority test; *P* < .05 indicates the noninferiority of MVC group compared with PICC. For all other comparisons, analyses intended to test superiority.

^d^ Number is descriptive; % cannot be provided.

^e^ Complete occlusion means that it is impossible to inject treatments and to draw blood. Partial occlusion means that it is impossible to draw blood.

Occlusion was the most frequent VC dysfunction. The complete occlusion rate was not significantly different between the MVC and PICC groups (7 of 135 [5.2%] vs 6 of 137 [4.4%]; *P* = .76), but partial occlusion was significantly more frequent in the MVC group than in the PICC group (30 of 135 [22.2%] vs 1 of 137 [0.7%]; *P* < .001). There was no significant difference in thrombophlebitis between the MVC and PICC groups (2 of 135 [1.5%] vs 1 of 137 [0.7%]; *P* = .62). There was no bloodstream infection in either group.

There were 15 and 8 VC-related interventions in the MVC and PICC groups, respectively (Table 3). VC replacement was required in 9 patients (6.7%) in the MVC group and in 7 patients (5.1%) in the PICC group (*P* = .59). PICC was chosen as the VC replacement for 15 patients (94.0%). Premature VC removal was significantly higher in the MVC group than in the PICC group (8 of 135 [6.0%] vs 1 of 135 [0.7%]; *P* = .02).

**Table 3.** Venous Catheter–Related Interventions at Follow-Up

	Patients, No. (%)		*P* value^a^
	MVC (n = 135)	PICC (n = 137)	
Patients with at least 1 VC-related intervention	9 (6.7)	7 (5.1)	.59
Total No. of VC-related interventions	15^b^	8^b^	
Anticoagulation for thrombophlebitis	1 (0.7)	1 (0.7)	>.99
Antibiotic therapy for VC infection	1 (0.7)	0	.50
Management of bleeding	2 (1.5)	0	.25
VC unclogging	1 (0.7)	0	.50
VC removal	1 (0.7)	0	.50
VC replacement	9 (6.7)	7 (5.1)	.59
New MVC	1 (0.7)	0	.50
New PICC single lumen	7 (5.2)	5 (3.7)	.54
New PICC 2 or 3 lumens	1 (0.7)	2 (1.5)	>.99
Reasons for VC replacement			
VC-related adverse event	2 (1.5)	2 (1.5)	>.99
VC dysfunctions	7 (5.2)	2 (1.5)	.10
Partial occlusion	3 (2.2)	1 (0.7)	
Total occlusion	2 (1.5)	0	
Migration	2 (1.5)	1 (0.7)	
VC limitations	0	2 (1.5)	>.99
Other^c^	0	1 (0.7)	.50
VC status at last follow-up			
Removed, end of treatment	94 (69.6)	105 (76.6)	.04
Removed due to VC dysfunction or adverse event	8 (5.9)	1 (0.7)	
VC still in place for treatment	33 (24.4)	31 (22.6)	
Patient lost to follow-up	0	0	
Removal status at last follow-up	134 (99.3)	137 (100)	
Premature removal due to VC dysfunction or adverse event	8 (6.0)	1 (0.7)	
No premature removal	126 (94.0)	136 (99.3)	.02

Abbreviations: MVC, midline venous catheter; PICC, peripherally inserted central catheter; VC, venous catheter.

^a^ Calculated with Pearson χ^2^ test, Fisher exact test, or Wilcoxon-Mann-Whitney test, as appropriate.

^b^ Number is descriptive; % cannot be provided.

^c^ Other reasons for VC replacement were unspecified.

## Discussion

This trial did not find MVCs to be a noninferior alternative to PICCs for 1 to 4 weeks of peripheral IVT in a tertiary care setting, mainly due to a significantly greater number of VC dysfunctions occurring with MVC. These dysfunctions resulted in a significantly greater rate of premature VC removal in the MVC group than in the PICC group (6.0% vs 0.7%; *P* = .02). Despite the exclusion criteria and protocol change to decrease the need for blood draws through the VC, many MVCs failed because their users wanted blood drawn. However, even after exclusion of partial occlusion from the analysis of the primary end point, in a direct comparison MVC could not be demonstrated as noninferior to PICC when used for infusion alone.

Although 3146 (46.1%) PICC requests were not eligible for MVC, according to the Infusion Nurses Society 2016 Therapy Standards of Practice,^1^ the other requests would have been eligible outside of the study protocol, confirming that a large proportion of PICC in a tertiary care center are prescribed for IVT that does not require a central VC. Other exclusion criteria in this study were not considered contraindications for MVC. The exclusion of 2081 (30.5%) patients likely to need a central VC at follow-up was intended to reduce the number of VC failures in the MVC group.

Blood sampling through a VC is generally recommended when the benefits outweigh the risks from increased hub manipulations and associated infection, occlusion, and erroneous laboratory values.^1^ In a tertiary care center, there are many patients with difficult blood draws for whom the VC is the only reasonable way to obtain blood sampling without prolonged, painful, and time-consuming venipuncture attempts. Increasing the number of repeated venipuncture attempts is not only painful for the patient but also mobilizes human resources and time that must be accounted for in the cost of an MVC, especially in an environment of a nursing staff shortage.

In this trial, while strict exclusion criteria reduced patient enrollment, they also reduced the percentage of patients with an adverse event or VC dysfunction in the PICC group (6.6%; 95% CI, 2.4%-10.7%) compared with the percentage in the literature and prior audits (20.0%).^6,8^ In the MVC group, the percentage of patients with adverse event or VC dysfunction remained high (33.3% [45 of 135]) but was comparable with the percentage in prior studies (13.2%-30.0%) despite their shorter VC dwell time.^6,8,11,17^

The comparison of MVCs and PICCs for peripheral IVT has been the subject of debate in the literature.^6,8,9,15,16,17,18,19^ MVCs may be a good option for ambulatory patients who do not need serial blood samplings.^9,18^ However, whether MVCs could replace PICCs in a tertiary care center is controversial.^8,11^ Retrospective studies comparing MVCs and PICCs for short-term antibiotic therapy reported contradictory results.^8,9,17,20^ A retrospective study including 367 patients from a general hospital reported a higher rate of complication with the use of MVCs compared with PICCs (19.5% vs 5.8%, *P* < .001).^17^ However, a retrospective multicenter study including 10 863 patients concluded that MVCs were associated with a lower risk of bloodstream infection and occlusion compared with PICCs.^9^ Differences in outcomes may be due to various selection bias in retrospective studies, which often miss important variables that were considered when choosing the VC and that may not be overcome by statistical adjustments. In addition, these retrospective studies may not have gathered clinical information, such as the need for blood sampling in patients with difficult venous access.^9^

To our knowledge, the only published randomized clinical trial comparing MVCs with PICCs in a tertiary care center reported significantly higher complication and premature VC removal rates in the MVC group as well as low rates of VC-related thrombophlebitis and infections that did not significantly differ between groups.^11^ The occlusion rate, however, was much lower in the previous trial than in the present study (2.0% vs 27.4% [complete and partial occlusion]). Differences in occlusion rates may be due to different inclusion and exclusion criteria and to potential selection bias; the number of patients excluded and the reasons for exclusion were not registered in that trial.^11^ The high rate of IVT extending beyond 30 days and the VC dwell time more than twice that reported in a previous trial^11^ are likely due to the tertiary care setting. Higher number of blood draw attempts through the VC may have uncovered the greater partial obstruction rate with MVC, likely due to its tip ending in a smaller, more collapsible vein, than with PICC (22.2% vs 0.7%). Venous collapse does not prevent VC injection, explaining why complete obstruction rates of MVC and PICC were comparable (5.2% vs 4.4%).

### Limitations

This trial has several limitations. First, there was a lower number of participants than required in the sample size calculation and a higher number of patients free from catheter-related adverse events or dysfunctions than estimated in the PICC group, limiting the statistical power of the study. However, given the importance of the greater dysfunction rate in the MVC group, noninferiority of these VCs as a substitute for PICCs in a tertiary care center is unlikely to be demonstrated with a larger number of participants. Second, the external validity of the results of this single-center study limits generalizability of the findings. Data on a small number of patient comorbidities were gathered, and vancomycin use was not excluded. Third, protocol changes during the research period could have introduced variability and affected the consistency of the study population and the estimate of the relationship between the VC type and the primary outcome. This trial aimed to assess whether an MVC could be an alternative when a PICC was requested for IVT that does not require a central VC in a tertiary care center. However, we did not address whether MVCs could be an appropriate option for IVT in other settings, wherein a greater proportion of outpatients do not require repeated blood sampling through the VC or prolonged IVT. Fourth, the open-label design of the trial could have led to performance and detection biases.

## Conclusions

In this randomized clinical trial in a tertiary care center, a substantial portion of PICCs were requested for peripheral IVT that would have been eligible for an MVC. Applying exclusion criteria to reduce the likelihood of MVC failure substantially decreased the number of patients who could receive an MVC. However, even in this select subgroup of patients, MVCs were associated with a significantly higher percentage of patients with a VC-related adverse event or dysfunction and a higher rate of premature VC removal compared with PICCs and could not be demonstrated as a noninferior alternative to PICCs.

## Notes

Supplement 1.
Trial Protocol

Supplement 2. **eTable.** Venous Catheter Related Adverse Events or Dysfunction, Excluding Partial Occlusion

Supplement 3.
Data Sharing Statement
