# Supplementary material for: Midline Venous Catheter vs Peripherally Inserted Central Catheter for Intravenous Therapy: A Randomized Clinical Trial
Source: JAMA Netw Open. 2025 Mar 20;8(3):e251258. doi: 10.1001/jamanetworkopen.2025.1258 (PMC11926630; doi:10.1001/jamanetworkopen.2025.1258)
Supplement: Supplement 1. — Trial Protocol [file jamanetwopen-e251258-s001.pdf]

Safety and efficacy of Midline and Peripherally Inserted Central Catheter for intravenous therapy: a randomized controlled trial

1.The Need for Trial

1.1 What is the Problem to be addressed?

Vascular access devices (VADs), including peripherally inserted central venous catheters (PICCs) remain a cornerstone for the delivery of necessary intravenous therapy. PICCs are being selected for venous access more frequently today than ever before. Furthermore, the proliferation of nurse-led PICC teams has made their use convenient and accessible in many settings (1, 2).

Peripherally inserted central catheters (PICCs) and midlines are non-permanent vascular access devices. These devices are both inserted above the antecubital fossa area but the position of the end of the catheter and the recommended dwell time differ. A PICC terminates in the superior vena cava (SVC), whilst a midline terminates in the axillary vein (3).

The Infusion Nurses Society standards of practice (2016) state that a PICC is classified as central access, indicated for irritating medications such as chemotherapy and some antibiotics due to greater hemodilution in the SVC (4). A midline is a peripheral device, appropriate for blood sampling and intravenous therapy with non-irritating medications such as antimicrobials, fluid replacement and analgesics with characteristics that are well tolerated by peripheral veins. Although midline can also be used with caution for intermittent vesicant administration (4) and the administration of vancomycin for less than 6 days, as was found to be safe in 1 study (5), a central catheter (such as a PICC) should usually be used for these medications for a longer period.

The advantage of PICC is that it can be used for all intravenous therapy. However, PICC requires the use of fluoroscopy or other type of guidance which add to the cost and time required to insert the catheter. Midline, on the other hand, can be inserted under ultrasound guidance, takes less time to be inserted and cost less than PICC insertion. The net savings obtained from placing the midline study device instead of a PICC was $90.00 per insertion (4).

Despite lower cost and better accessibility of midline in comparison with PICC for non-vesicant intravenous therapy, there is very little evidence in the literature to suggest that one type of venous access is better than the other. This evidence is based mostly on comparison of historical cohort series with various patient populations who were probably not comparable, and most of the literature recommendations are based on expert opinions with conflicting guidelines (6).

1.2 What are the principal research questions to be addressed:

HYPOTHESIS

The safety and efficacy of Midline is not inferior to PICC for specified indications.

PRIMARY OBJECTIVE

To assess the non-inferiority until 1 week after VAD retrieval for safety and efficacy of using Midline in comparison with PICC for intravenous therapy that do not require a central catheter (recommendations of the infusion nurses society 2016 (4)).

SECONDARY OBJECTIVES

Compare between Midline and PICC:

A) The VAD-related adverse events rate.
B) The number of VAD-related adverse events/1000 catheter-days.
C) The time to first VAD-related adverse event.

D) The duration of intervention for VAD insertion.
E) The number of additional interventions to insert the VAD
F) The percentage of patients who required another VAD to complete the iv treatments
G) The percentage of patient without failed blood sampling attempts through the VAD.
H) The percentage of patients for whom the end of treatment was the reason for VAD retrieval.

1.3 Why is a trial needed now? Evidence from the literature - see 1.4 below, professional and consumer consensus and pilot studies should be cited if available.
There are a large number of prospective series showing that midlines are appropriate for medium-term intravenous antibiotics. However, there are very few non-controlled studies comparing PICC to Midline. One retrospective study comparing PICCs and MCs in 367 patients concluded that "MCs were associated with a higher risk of non–life-threatening complications versus PICCs, which showed fewer but more serious complications, including bacteremia. The decision to move toward more use of MCs is not without risk. Institutions should continue to review the utilization and safety data of IV catheter use to determine the most appropriate use of these devices" (7). Another study randomized PICC to peripheral catheter (not Midline) in 60 patients and concluded that "PICC is efficient and satisfying for hospitalized patients requiring i.v. therapy $\geq$ five days. However, the risk of DVT, mostly asymptomatic, appears higher than previously reported, and should be considered before using a PICC" (8). However, a prospective, randomized controlled trial including a total of 100 acute hospitalized patients with difficult venous access randomized to either short or long peripheral catheter reported a lower risk of catheter failure with the long peripheral catheter (9). Another retrospective observational study comparing Midline and PICC in 64 patients with cystic fibrosis concluded that "Midlines may be an alternative to PICCs for adult CF patients although further research is required with a larger sample size to enable definitive conclusions" (3). Finally, there is only one small randomized trial directly comparing Midline to PICC in a total of 54 patients but only in the context of short term vancomycin administration (5). That study, however, concluded that "short-term intravenous vancomycin can be safely and cost-efficiently administered in the deep vessels of the upper arm using the midline study device".

1.4 Give references to any relevant systematic review(s)1 and discuss the need for your trial in the light of the(se) review(s). If you believe that no relevant previous trials have been done, give details of your search strategy for existing trials.
Systematic reviews reported that Midlines are appropriate for medium term intravenous antibiotics (1, 3-5). The lack of controlled trials with large sample size brings a low level of evidence.

1.5 How will the results of this trial be used? E.g. inform decision making/improve understanding.
Results of this trial will allow improving a quality of evidence for using midline instead of PICC for specified indications. If Midline are non-inferior for some indications, they would represent a more accessible and less expensive alternative than PICC insertion.

1.6 Describe any risks to the safety of participants involved in the trial.
Risks associated to the trial are related to those of venous and central venous catheters and include venous thrombosis, premature dysfunction of the venous access and risk of central line-associated bloodstream infection (CLABSI) (4). If the nursing staff are not well informed, there is also a risk for the patient with a midline to receive a medication in the exclusion list. These risks are not different whether the patient participates or not in the trial.

2. The Proposed Trial

2.1 What is the proposed trial design? E.g. Open-label, double or single blinded, etc.

This will be a prospective randomized open-label parallel group clinical trial, in which safety and efficacy of Midlines will be compared to PICC in indications that are generally accepted for Midlines in the literature.

2.2 What are the planned trial interventions? Both experimental and control.

<u>MIDLINE:</u> Device insertion will be performed by the same staff who will insert PICCs: interventional radiologists, fellows and residents in interventional radiology, in the same angio suites used for PICC insertion, to minimize methodological bias between the 2 groups. The vein will be punctured under ultrasound guidance, in sterile conditions, a few centimeters above the elbow joint, at the same site where a PICC would be inserted. Disinfection and draping will be similar to PICC insertion. The 20 cm long, 4F, open end, single lumen Midline catheter, without valve (Bard PowerMidline catheter) will be inserted into the basilic or brachial vein without fluoroscopy. In case of insertion difficulties, attempt will be first performed in a another vein in the same arm and if unsuccessful, in the other arm. In rare case, if no insertion is possible, a MidLine will be installed under fluoroscopy. Distance between the axilla and the puncture site will be measured. The catheter will be cut if its tip is beyond the axilla so that it does not terminate in a curved venous segment. Although cutting a PICC to the desired length (20 cm) could serve as a Midline (and would decrease device bias between groups), PICCs have external identification as a "PICC" and their use as a Midline would be a source of error if ever other medications that require a central line would be needed. Therefore, we will use a 4F single lumen open end, Midline catheter without valve from the same company providing the PICC catheter. Catheter fixation and dressing will be similar for both group (Midline and PICC) at insertion and until catheter removal.

<u>PICC:</u> Peripherally inserted central catheters will be inserted by the same radiologists, fellows and residents who insert midlines, in the angiography suite. Insertion technique will be similar to midline insertion except that PICC (Bard PowerPICC) length will be individually tailored to be placed in the superior vena cava under fluoroscopy. Identification of the type of catheter inserted, whether it is a midline or a PICC, is shown on the catheter. The radiologist, fellow or resident who will insert the catheter will complete a preprinted order identifying the type of catheter installed and its length.

2.3 What are the proposed practical arrangements for allocating participants to trial groups? E.g. Randomization method. If stratification or minimization are to be used, give reasons and factors to be included.

Patients will be randomized to the following groups: A) PICC and B) Midline catheter.

Randomization will be stratified according to the indication for VAD: a) for antibiotics versus b) for other iv treatments as well as whether they a) have cystic fibrosis (CF) or b) do not have cystic fibrosis, given that these two factors may expose to thrombosis (3,5).

Allocation will be done by "Blocked Randomization", with randomly varying block sizes between 2 and 4 (so that the maximum difference between the total number of patients randomized in both 2 groups will not be greater than 2 (half of the largest bloc). Small bloc sizes will allow to minimize unbalanced size of both arms of the study in each stratum. With random variation of block size, it will be impossible, for the research staff (physician, patients etc), to predict the treatment group to which the next patient will be randomized. The REDCap randomization tool will be used to facilitate randomization. Randomization allocation tables will be generated by a statistician using R software (package blockrand), and will be uploaded into the REDCap project, or will provide to the REDCap Administrator to be uploaded. The

statistician will generate the random allocation tables according to study design specifications. Participants will be randomized when research nurses enter a participant’s REDCap record and click the “Randomize” button. Clicking this button triggers REDCap to check the allocation table according to the stratification variables and display the group to which the participant should be randomly assigned. This assignment is permanent and not editable within the participant record and, like all other activity within REDCap, is tracked and not modifiable in the audit log.

2.4 What are the proposed methods for protecting against sources of bias? E.g. Blinding or masking. If blinding is not possible please explain why and give details of alternative methods proposed, or implications for interpretation of the trial's results.

The patient and the medical team (referring physicians and nurses) treating the patient will know which type of catheter has been installed in radiology. It is difficult to safely hide the randomization group to the medical team (physician and nurses) because the operator needs to know which kind of venous access will be used and medical team needs to know what kind of venous access the patient has because a midline may not be appropriate if there is a change of intravenous medications after the venous access has been selected (which medication could require a central access). It is also important that appropriate care is brought to both Midline and PICC. However, to prevent any technical bias, patients in group I (Midline) will be prepared and positioned the same way as those in group II (PICC) in the angiography room.

2.5 What are the planned inclusion/exclusion criteria?

All consecutive patients, hospitalized or not, who are referred to the radiology department for a midline or a PICC line will be considered for inclusion in this trial if they meet the following inclusion/exclusion criteria and accept to participate to the study.

<u>Inclusion criteria:</u>

Age > 18 years

Required intra venous therapy

Expected duration of the venous access: > 6 days and < 30days,

<u>Exclusion criteria:</u>

Patient has a contraindication to insertion of either a Midline or a PICC

A) Relative to the therapy: NB: A list of medication (Cf annex 1) which can be given through a peripheral line (Midline) will be kept up to date by the pharmacy of the CHUM. Support from the pharmacy of the CHUM (Ms. Pascaline Bernier, extension 36251 or through the pager 8444) will be provided throughout the study. The pharmacy will also be aware of the kind of catheter that was inserted in the patients participating in this study to prevent administration of drugs inappropriate for a midline. A patient entry in the pharmacy software will allow to automatically show in the electronic medical record (Oacis) and in the registered drug form (FEM) that the patient has been enrolled in the protocol and which kind of catheter was inserted.

Vesicant therapy,

    pH less than 5 and greater than 9
    Infusates with an osmolarity greater than 900 mOsm/L, (ex: Dextrose > 10 %)
    Parenteral nutrition
    Chemotherapy
    Potassium >40 meq/L

Vasopressors: Dopamine dobutamine noradrenaline phenylephrine

B) Relative to the patients:

Patient from other hospitals who come to the CHUM only for the installation of a central line

Decreased cognitive ability to care for device at home
Preexisting venous thrombosis or known hypercoagulable states (such as protein C or S deficiency, antithrombin deficiency, lupus anticoagulant)
End-stage renal disease requiring vein preservation (4).
Venous access with multiple lumens required
Patients not able to give informed consent
Prior participation to this study
Patient is enrolled in another investigational study

2.6 What is the proposed duration of treatment period?
Although the intent to treat duration of venous access insertion is expected to be > 6 days but < 1 month a priori, these accesses may be used for a longer period depending on clinical needs. Decision about catheter retrieval will be made according to the medical team.

2.7 What is the proposed frequency and duration of follow up?
All patients will be followed until the venous access is removed. The hospitalized patients will have a weekly clinical monitoring until one week after the venous access is removed. Outpatients will have a weekly telephone call until one week after the venous access is removed.

2.8 What are the proposed primary and secondary outcome measures?
<u>Primary safety outcome:</u> Percentage of patients without VAD-related adverse event (complications and VAD dysfunction) requiring medical intervention (including but not limited to VAD removal/repositioning/replacement) during follow-up (until 1 week after VAD retrieval):
1-Complications
a) Suspected or confirmed infections -Catheter-related blood stream infection (CR-BSI),
-Local infections

b) Thrombophlebitis -Deep vein thrombosis (DVT),
-Superficial phlebitis (thrombotic or chemical)

c) Infiltration
d) Pain
e) Bleeding
f) Death

2-VAD dysfunctions
a) Accidental withdrawal or migration
b) Leakage or fracture
c) Obstruction (partial or complete) -impossible to inject required treatments
-impossible to draw blood
<u>Secondary safety outcomes</u>:
a) Percentage of patients who experience each of the above-mentioned events requiring medical intervention (including but not limited to VAD removal/repositioning/replacement) during follow-up (until 1 week after VAD retrieval).
b) Number of the above-mentioned events requiring medical intervention (including but not limited to VAD removal/repositioning/replacement) per 1000 catheter/days.
c) Time to first VAD-related adverse event.

<u>Primary efficacy outcome:</u> Percentage of patients whose intended or additional intravenous treatments could be completed with the VAD without limitations (choice of drug, posology and

duration of treatment) due to VAD complications, dysfunctions or limitations (ex: need for central access or need for additional peripheral or central lumens/catheters).

Secondary efficacy outcomes:
Immediate
a) Duration VAD intervention (Midline or PICC).
b) Number of additional interventions to insert the VAD (Contrast injection, vein dilation or recanalization, fluoroscopy required, failed attempt requiring to access the other arm).
At follow-up
a) Percentage of patients who required another VAD to complete the intended or additional iv treatments, either because of VAD complications or VAD dysfunctions or VAD limitations
b) Percentage of patient without failed blood sampling attempts through the VAD.
c) Percentage of patients for whom the end of treatment was the reason for VAD retrieval.

2.9 How will the outcome measures be measured at follow up?
Assessment of efficacy
Every week following VAD insertion, the research nurse will communicate with the nurse in charge of the patient when hospitalized or at the CLSC and directly with the patient when the patient has no regular visit at home. Efficacy will be assessed by the number of events as defined in point 2.8

Assessment of safety
Assessment of infection
This study used a diagnostic definition of CR-BSI, which aims to identify the VAD as the specific source of infection. New acute fever will be investigated by culture of two blood samples (at least one drawn by venipuncture) and if the catheter is the suspected source of infection, it will be removed and cultured. Confirmed catheter-related BSI will be defined as the association of a positive blood culture in a patient having had a central line within 48 h prior to the onset of symptoms, AND one of the following criteria: 1) a positive culture of either catheter tip or exit site swabbing (≥103 CFU/ml) involving the same organism as blood culture, 2) blood cultures from peripheral venous puncture and central lines positive with the same organism with a quantitative ratio (central sample/peripheral sample) > 5, or 3) a differential time to positivity > 2 h in favor of central line sample.
Catheter-related soft tissue infection will be diagnosed when erythema, induration, and pus will be present at the site of insertion, in the presence of clinical or biological sign of infection. Very localized exit site erythema, as often observed with PICCs or PCs, will not be considered as a soft tissue infection. Confirmed catheter-related local infection (LI) will be defined as a positive culture of the PICC segment (≥103 CFU/ml) with pus emerging from the exit site or a tunnel infection, with local manifestations of infection but no general signs of sepsis and negative blood cultures.
When all these criteria are not present or bacteriological culture not realized, or realized when the patient was under antibiotic therapy, suspected infections will be classified as “possible infection”. When cultures remain negative (in the absence of antibiotics) or another cause of infection is diagnosed, the case will be classified as “infection not confirmed”.

Assessment of thrombophlebitis
Deep venous thrombosis: Patients who report local pain, superficial vein enlargement or upper limb edema will be referred for Duplex ultrasound examination of superficial and deep veins of the upper limb, by compression ultrasonography (CUS). In addition, proximal venous flows will be assessed to detect indirect signs of central venous thrombosis. Catheter-related venous

thrombosis will be defined by the presence of non-compressible material in the vein lumen. Thromboses of the subclavian, axillary, and humeral veins will be classified as deep vein thrombosis DVT, whereas thromboses of the basilic and cephalic veins will be classified as superficial phlebitis (thrombotic or chemical). Phlebitis will be assessed according to a standardized phlebitis scale reported in Annex 2 (Table 1 page S96 of ref 4).

Occlusion, pain, bleeding, infiltration, phlebitis, catheter fracture, leakage and dislodgement will be included when documented in the medical record by a healthcare professional, whether medical or nursing staff.

2.10 Will health service research issues be addressed? Justify inclusion/exclusion of health economics and quality of life measures. If these measures are to be included full details should be given including power calculations.

There will be no treatment cost analysis nor cost-benefit assessment in this project given that it is essentially a study about efficacy and safety of VADs. In addition, several studies have shown the lower cost of Midline in comparison to PICC.

2.11 What is the proposed sample size and what is the justification for the assumptions underlying the power calculations? Include both control and treatment groups, a brief description of the power calculations detailing the outcome measures on which these have been based, and give event rates, means and medians etc. as appropriate.

Sample size determination is based on the primary safety outcome: Percentage of patients without VAD-related adverse event requiring medical intervention during follow-up. Given the higher cost and lesser availability of PICC in comparison to Midline, we would consider that a Midline would be a better option if it is non-inferior to a PICC in situations where a midline could be used. There is a great variability in reported VAD complications, dysfunctions and limitations in the literature and there are no controlled study comparing Midlines to PICCs for iv treatment up to 4 weeks. In a prior study about 1273 PICCs (10) 25% of patients required more than 1 PICC to complete the treatment. In another study about 393 PICCs (11), 21% of PICCs must be retrieved before the end of the treatment. Another study of 322 PICCs reported, that 41% of PICCs were retrieved for a reason other than the end of the treatment (12). A prior audit of 192 PICCs inserted at the CHUM reported 20.3% (39/192) infections (most were "suspected"), 13% (25/192) technical problems and 3.1% (6/192) thrombophlebitis. However, complications are expected to be lower for VAD that would be suited for midline (no vesicant drugs, lower proportion of cancer patients and shorter treatment duration). Based on these results from our experience and from the literature, average percentage of patients without VAD-related adverse events was conservatively assessed to be 75%. With a noninferiority margin set at 10%, which corresponds to an adverse event relative risk of 1.4 for the group receiving Midline, 232 patients per group would be needed to have 80% power (beta) to exclude a difference in favor of the PICC group of more than 10% over midline, with a 0.05 one-sided significance level (Sealed Envelope Ltd. 2012. Power calculator for binary outcome non-inferiority trial. [Online] Available from: https://www.sealedenvelope.com/power/binary-noninferior/ [Accessed Mon Feb 26 2018].). To compensate for patient withdrawal and lost to follow-up, the sample size is increased by 10% to 510 patients (255 patients per group).

2.12 What is the planned recruitment rate? How will the recruitment be organized? Over what time period will recruitment take place? What evidence is there that the planned recruitment rate is achievable?

Most VAD request in our center are for PICC although nearly half of these would be suited for Midline according to the present inclusion/exclusion criteria. We perform about 10-12

PICCs daily and, therefore, 3-5 PICCs could be enrolled in the study per day. With a conservative inclusion rate of 3 PICCs per day, study enrollment and patient follow-up should be completed within 9 and 10 months respectively.
The research nurse will screen all requests for Midline and PICC in the Radiology Information System (RIS) and will verify whether the patient is eligible to the study. The research nurse will evaluate each participant hospital file to be sure that each criterion is met, including the registered drug form (FEM) available at the nurse stations for inpatients. The research nurse will also talk to the nurse assistant or the patient's nurse to be sure that, in the following days, there is no planned treatment not written in the FEM that would be a contraindication for the installation of the midline. Informed consent will be obtained the same day as the VAD request for inpatients while outpatients will be contacted by telephone before the day of intervention and the informed consent form will be signed the day of the intervention.

2.13 Are there likely to be any problems with compliance? On what evidence are the compliance figures based?
Most patients who will be hospitalized and we expect no problem of compliance for these patients given that they have no specific action to take after VAD insertion. To prevent compliance problems in ambulatory patients, a letter presenting the project will be annexed to the "requête inter-établissement" (DSIE) so to inform community nurses, who might be responsible for catheter care and surveillance, about the specificity of the project.

2.14 What is the likely rate of loss to follow up? On what evidence is the loss to follow-up rate based?
Loss to follow-up should be low given that most patients are hospitalized during the whole duration of their iv treatment. Lost to follow-up may be a little higher for outpatients but should remain low given that the follow-up questionnaire is relatively straightforward. Given that the research nurse will offer to see the patient in case of suspected complication or catheter dysfunction, we expected the loss to follow-up rate to be below 10%.

2.15 How many centers will be involved?
All patients will be selected from requests addressed to the radiology department of CHUM.

2.16 What is the proposed type of analyses?
Proportions of events will be compared using the Fisher exact test. Continuous variables will be compared by the Student's t-test or Mann–Whitney rank sum test according to normality of their distribution. Safety and efficacy outcomes will be analyzed using logistic regression including terms for treatment group and randomization stratification factors as well as other demographic, treatment and time variables. . Time-to-event outcomes will be evaluated using Kaplan-Meier curve and cox regression. A switch of treatment (midline to PICC or PICC to midline) will be consider as an event.

Analysis will be performed with both the ITT paradigm and the per-protocol (PP) analysis set. The intent-to-treat (ITT) principle states that all randomized patients are analyzed according to the treatment to which they were randomized. This analysis is intended to avoid various biases associated with patients switching treatment, selection bias, and dropout/withdrawal patterns that may confound the observed treatment effect. This is recognized as a potentially conservative analysis in superiority trials.

In non-inferiority trials however, many kinds of problems fatal to a superiority trial, such as non-adherence, misclassification of the primary endpoint, or measurement problems or many dropouts who must be assessed as part of the treated group, can bias toward no treatment difference (success) and undermine the validity of the trial, creating apparent non-inferiority where it did not really exist.

We will thus perform, additionally to an ITT analysis, a per-protocol (PP) analysis, where the PP analysis set is defined by the treatment (PICC or Midline) the patient had really. Differences in results using the two analyses (ITT and PP) will need close examination and both analysis will be reported.

2.17 What is the proposed frequency of analyses?
Analyses will be completed once, at the end of the study. There will be no interim analysis given that both treatment modalities are well accepted in the literature and that the study will be completed within a year.

2.18 Are there any planned subgroup analyses?
The adverse event rate is expected to be higher for "antibiotherapy indication" than for "indication other than antibiotherapy" and in patients with, rather than without, cystic fibrosis, justifying stratification of the randomization according to the indication of the VAD and diagnostic of cystic fibrosis (see section 2.3). However, it is uncertain whether VAD safety and efficacy will vary according to the indication for VAD and the presence or absence of cystic fibrosis. Therefore, we propose to first test the interaction test between the two binary variables representing, respectively, the two treatment groups (Midline vs PICC) and 1- the type of indication and 2- the presence or absence of cystic fibrosis. This interaction will be included and tested in each of the multivariable models proposed for respective outcomes in section 2.16. If the null hypothesis of no interaction is rejected at a 2-tailed $\alpha=0.05$, we will proceed with subgroup analyses, in which the effect of VAD type on a given outcome will be estimated and tested separately for each of the two indications for VAD and for the presence or absence of cystic fibrosis. In contrast, a non-significant interaction will be removed from the final model and in this case, we will report the overall VAD type effect using data pooled from subjects with both types of indication for VAD, with or without cystic fibrosis. This approach, where subgroup analyses are conditional to the significance of the interaction, is recommended to reduce the risk of spurious findings, due to inflated type I error (13).

2.19 Has any pilot study been carried out using this design?
There will be no pilot study. However, the protocol will be tested with 5 roll-in patients before the start of the study.

3. Trial Management
3.1 What are the arrangements for day to day management of the trial? E.g. Randomization, data handling, and who will be responsible for coordination.
The clinical database will be centralized by Ms Jennifer Satterthwaite, research coordinator of the radiology department (CHUM). The database will be confidential with a secured access. The CHUM research assistants involved in data entry (Line Julien and Assia Belblidia) have their Good Clinical Practice credential. Online software will generate the randomization lists separated for each stratum (Sealed Envelope Ltd. 2017. Create a blocked randomisation list. [Online] Available from: https://www.sealedenvelope.com/simple-randomiser/v1/lists [Accessed 16 Feb 2018].). Based on these lists, Ms Satterthwaite will prepare 4 series of

sealed envelopes identified as 1- "indication antibiotic, patient with cystic fibrosis" 2- "indication antibiotic, patient without cystic fibrosis", 3- "indication other than antibiotic, patient with cystic fibrosis" and 4- "indication other than antibiotic, patient without cystic fibrosis". In each series, envelopes will be identified from 1 to 300, corresponding to the enrollment order of patients into the strata. Individual envelopes with treatment allocation of consecutive patients will be prepared centrally, by personnel not involved in the trial. The envelopes will be opened only when the patient is found eligible and has signed the informed consent form. The envelop will contain a card indicating the assigned treatment the patient was randomized to. Ms Satterthwaite will keep the master copy of the lists. Stratification by indication (antibiotics or not) for VAD and by presence or absence of cystic fibrosis will reduce the risk of imbalance, between the two trial arms, in the distribution of these important factors. In each of the 4 strata, subjects will be assigned to Midline or PICC using blocked randomization, with random block sizes of 2 or 4.

3.2 What will be the role of each principal applicant and co-applicant proposed?

Dr Eric Therasse is an interventional radiologist at the Centre Hospitalier de l'Université de Montreal (CHUM) and professor of Radiology at University of Montreal (UoM). As the PI, of this study, he will assume the scientific and administrative coordination of the project. He will be responsible for the data analysis and for publication of the results.

Dr Ahmed Bentridi is a radiologist in Algeria and fellow in interventional radiology in the CHUM. He contributed to the study design and will coordinate the clinical follow-up in the CHUM. He will participate to the data analysis and he will write and publish the manuscript.

Dr Gilles Soulez is an internationally known interventional and vascular radiologist, Professor of Radiology at UoM and holder of a National Researcher Award (FRSQ). He is Academic Chair and Director of Research, Department of Radiology at UoM. He contributed to the design of the study. He will participate to the patient enrolment and to the HA interventions in the CHUM. He will participate to the data analysis, to the manuscript writing and to the revision of the final paper.

Ms Audrey Chouinard, M.Sc (CSIO) Clinical nurse specialist in oncology, Centre intégré de cancérologie, DSI-RC is a nurse involved in oncology and member of the venous access committee of the CHUM. She contributed to the design of the study as well as to the safety of the conduct of the trial. She will participate in the date analysis and the manuscript writing.

Pascaline Bernier is a pharmacist at the Centre d'information du CHUM. She participated to the study design and for the coordination of the clinical follow-up. She contributed to the improvement of the definition of the drugs that will be allowed in this trial and to the design of the whole study. She will be an important resource for all pharmacological and methodological considerations. She will also participate to the data analysis and to the revision of the final manuscript.

Dr Vincent Oliva is an internationally recognized interventional radiologist. He is the chief of the department of radiology of the CHUM. He contributed, to the definition of the safety and efficacy issues and to the design of the whole study. He will supervise patient enrolment and participate to the data analysis and manuscript writing.

Dr Patrick Gilbert is a young interventional radiologist at the CHUM. He will supervise patient enrolment and perform VAD insertion in randomized patients. He will participate to the data analysis and to the manuscript writing.

Dr Pierre Perreault is an interventional radiologist at the CHUM. He contributed to the study design and will participate to the patient enrolment. He will participate to the data analysis and manuscript revision.

Dr Louis Bouchard is an interventional radiologist at the CHUM. He will supervise patient enrolment and perform VAD insertion in randomized patients.

3.3 Describe the trial steering committee and if relevant the data safety and monitoring committee.

Steering committee: Dr E. Therasse, Audrey Chouinard, Pascaline Bernier and G. Soulez. There will be no data safety and monitoring committee given that this trial involves interventions that are well accepted for approved indications.

3.4 Ethical Considerations

All the information collected during the research project will remain strictly confidential to the extent provided by law. Data collection will be centralized. This database will be confidential with subject code numbers and a securized access limited to LCTI research assistants and archived in the CRCHUM network. Data will be entered by trained site personnel in RedCap database with reasons given for any missing data. Any errors should be corrected within the electronic system. The audit trail will record all changes made, the date and time of the correction, and the person correcting the error and the reason for the correction. The appropriate electronic signature will be provided. Any data recorded directly in the eCRF, for which no other written or electronic record will be maintained in the patient's research file, will be considered source data and should be signed by the Investigator(s).The research assistants involved in the data collection (L. Julien, J.Satterthwaite, Casey Bourdeau Caporuscio) are accredited according to the good clinical practice for research norms. The research assistants will be responsible for the processing, quality control and management of the data.The principal investigator will only collect information required to meet the scientific goals of the study. The study data may be published or shared during scientific discussions; however it will not be possible to identify the subjects.

All data will be securely stored in paper (binders in a locked cabinet) or electronic format (CRCHUM network) for 10 years by the principal investigator.
