# Supplementary material for: Midline Venous Catheter vs Peripherally Inserted Central Catheter for Intravenous Therapy: A Randomized Clinical Trial
Source: JAMA Netw Open. 2025 Mar 20;8(3):e251258. doi: 10.1001/jamanetworkopen.2025.1258 (PMC11926630; doi:10.1001/jamanetworkopen.2025.1258)
Supplement: Supplement 2. — eTable. Venous Catheter Related Adverse Events or Dysfunction, Excluding Partial Occlusion [file jamanetwopen-e251258-s002.pdf]

Supplemental Online Content

Bentridi A, Giroux M-F, Soulez G, et al. Midline venous catheter vs peripherally inserted central catheter for intravenous therapy: a randomized clinical trial. *JAMA Netw Open*. Published online March 20, 2025. doi:10.1001/jamanetworkopen.2025.1258

eTable. Venous Catheter Related Adverse Events or Dysfunction, Excluding Partial Occlusion

This supplemental material has been provided by the authors to give readers additional information about their work.

© 2025 Bentridi A et al. *JAMA Network Open*

eTable. Venous Catheter Related Adverse Events or Dysfunction, Excluding Partial Occlusion

	MVC (n= 135)	PICC (n= 137)	P value†
**Patients without VC-related dysfunction or adverse event 95%CI**	119 (88.1) (82.7-93.6)	129 (94.2) (90.2-98.1)	**.13‡**
Patients with at least 1 VC-related dysfunction or adverse event			
Patients with 1 VC- related dysfunction or adverse event	13 (9.6)	7 (5.1)	0.23
Patients with 2 VC- related dysfunction or adverse event	3 (2.2)	1 (0.7)	
VC-related dysfunction or adverse event (total number)	19	9	
Catheter dwell time (days)			
Median (Q_1-Q_3)	26 (21-36)	29 (22-37)	.40
Total number of catheter-days	3,813	4,070	-
VC-related dysfunction or adverse event per 1,000 catheter-days	5.0	2.2	0.04
**Patients without VC-related dysfunction**	124 (91.9)	130 (94.9)	0.32
Patient with related dysfunction	11 (8.1)	7 (5.1)	0.32
Patients with 1 VC- related dysfunction	10 (7.4)	7 (5.1)	0.39
Patients with 2 VC- related dysfunction	1 (0.7)	0 (0.0)	
VC-related dysfunction (total number)	12	7	
VC-related dysfunctions per 1,000 catheter-days	3.1	1.7	0.20
Patients with VC migration or accidental withdrawal	4 (3.0)	1 (0.7)	.21
per 1,000 catheter-days	1.0	0.2	.19
Patients with VC complete occlusions	7 (5.2)	6 (4.4)	.76
per 1,000 catheter-days	1.8	1.5	.69
**Patient without VC-related adverse event**	128 (94.8)	135 (98.5)	.10
Patient with 1 VC- related adverse event	7 (5.2)	2 (1.5)	
VC-related adverse event (total number)	7	2	
VC-related adverse event per 1,000 catheter-days	1.8	0.5	.10
Patients with VC infiltrations	1 (0.7)	0 (0.0)	.50
per 1,000 catheter-days	0.3	0.0	>.99
Patient with VC bleedings at insertion site	3 (2.2)	0 (0.0)	.12
per 1,000 catheter-days	0.8	0.0	>.99
Patient with VC-related infections	1 (0.7)	1 (0.7)	>.99
per 1,000 catheter-days	0.3	0.2	.96
Patients with local infections	1 (0.7)	1 (0.7)	>.99
per 1,000 catheter-days	0.3	0.2	.96
Patients with blood stream infections	0 (0.0)	0 (0.0)	-
per 1,000 catheter-days	0.0	0.0	-
Patient with VC-related thrombophlebitis	2 (1.5)	1 (0.7)	.62
per 1,000 catheter-days	0.5	0.2	.54
Patients with deep thrombophlebitis	0 (0.0)	0 (0.0)	-
per 1,000 catheter-days	0.0	0.0	-
Patients with superficial thrombophlebitis	2 (1.5)	1 (0.7)	.62
per 1,000 catheter-days	0.5	0.2	.54

Results presented are N (%) unless stated otherwise.
‡ Non-inferiority test, a p-value less than 5% would indicate the non-inferiority of MVC group compared to PICC. For all other comparisons, analyses intent to test superiority
† Pearson Chi-Square test, Fisher's exact test, Wilcoxon Mann-Whitney test, as appropriate.

© 2025 Bentridi A et al. *JAMA Network Open*

Wilcoxon Mann-Whitney test was used for comparison of medians. Poisson regression was employed for comparison of rates per 1,000 catheter-days.
* Complete occlusion = impossible to inject treatments and to draw blood
VC, venous catheter, PICC, peripherally inserted central catheter; MVC, Midline venous catheter CI, confidence interval; Q1, first quartile; Q3, third quartile

© 2025 Bentridi A et al. *JAMA Network Open*
