# Supplementary material for: Midline Venous Catheter vs Peripherally Inserted Central Catheter for Intravenous Therapy: A Randomized Clinical Trial
Source: JAMA Netw Open. 2025 Mar 20;8(3):e251258. doi: 10.1001/jamanetworkopen.2025.1258 (PMC11926630; doi:10.1001/jamanetworkopen.2025.1258)
Supplement: Supplement 3. — Data Sharing Statement [file jamanetwopen-e251258-s003.pdf]

Data Sharing Statement

Bentridi. Midline Venous Catheter vs Peripherally Inserted Central Catheter for Intravenous Therapy. *JAMA Netw Open*. Published March 20, 2025.
doi:10.1001/jamanetworkopen.2025.1258

Data
Additional Information: ClinicalTrials.gov identifier: NCT03502980
Data available: Yes
Data types: Deidentified participant data
How to access data: Data generated or analyzed during the study are available from the corresponding author by request.
When available: With publication

Supporting Documents
Document types: Statistical/analytic code
How to access documents: Data generated or analyzed during the study are available from the corresponding author by request.
When available: With publication

Additional Information
Who can access the data: Data will be made available to researchers whose proposed use of the data has been approved.
Types of analyses: Data will be made available for any purpose.
Mechanisms of data availability: Data will be made available without investigator support, with a signed data access agreement.
